# A Case of Special Complication following a Large Amount of Polyacrylamide Hydrogel Injected into the Epicranial Aponeurosis: Leukocytopenia

**DOI:** 10.1155/2015/695359

**Published:** 2015-10-20

**Authors:** Li Rong, Shi-Jie Lan, Ying Shao, Zhe Chen, Duo Zhang

**Affiliations:** ^1^Department of Plastic and Aesthetic Surgery, First Hospital of Jilin University, Chaoyang District, Changchun, Jilin 130021, China; ^2^Department of Hematology and Oncology, First Hospital of Jilin University, Chaoyang District, Changchun, Jilin 130021, China

## Abstract

Polyacrylamide hydrogel (PAAG) has been used as an injectable filler for soft tissue augmentation of different body parts, such as the face, breasts, and penis. However, this is the first report of leukocytopenia after injection of a large amount of PAAG in the epicranial aponeurosis. After receiving PAAG injection for craniofacial contouring, the female patient described herein experienced recurrent swelling, temporal pain (particularly with changes in ambient temperature and facial expression), and ultimately leukocytopenia due to widespread migration of the injected PAAG. We removed most of the PAAG from the affected tissues and the leukocytopenia disappeared 1 year after the operation. Based on this case, we hypothesize that injection of a large amount of PAAG into tissues that have ample blood supply, such as the epicranial aponeurosis, may induce leukocytopenia.

## 1. Background

Polyacrylamide hydrogel (PAAG) was introduced as a filler material for soft tissue augmentation in 1997. Different subtypes of polyacrylamide gel (PAAG) exist (Aquamid, Interfall, Outline, Formacryl, Bioformacryl, Argiform, Amazing Gel, and Bio-Alcamid). The gel consists of a minor backbone of 2.5% to 5% cross-linked polyacrylamide and 97.5% to 95% water, which accounts for its high biocompatibility [1]. Although the application of PAAG as a filler material was banned by most countries during the following decade, cases of complications related to PAAG injection still occasionally occur. PAAG was originally considered to be a safe biomaterial because it exhibits good tissue compatibility and elicits a weak inflammation reaction. However, due to its liquid state at physiological temperature, unintended spreading of PAAG is a common complication of this procedure. Furthermore, complete removal of the liquid filler material from the affected tissues is impossible. In this report, we present a case of widespread migration of PAAG following a large injection into the epicranial aponeurosis that induced recurrent swelling, temporal pain, and leukocytopenia and discuss the possible causes for these complications.

## 2. Case Presentation

A 50-year-old woman had undergone craniofacial augmentation involving bilateral injection of a large amount of PAAG into the temporal epicranial aponeurosis in 1997. During the 6 months after the operation, the patient complained repeatedly of recurrent swelling of the affected regions. To alleviate the symptoms, the plastic surgery clinic where she underwent the augmentation attempted to squeeze out the filler material through a 5 cm coronal incision in the scalp. The volumes of PAAG originally injected and then later removed are both unknown. After the operation to remove the PAAG, the patient's symptoms did not improve.

**Figure 1:**
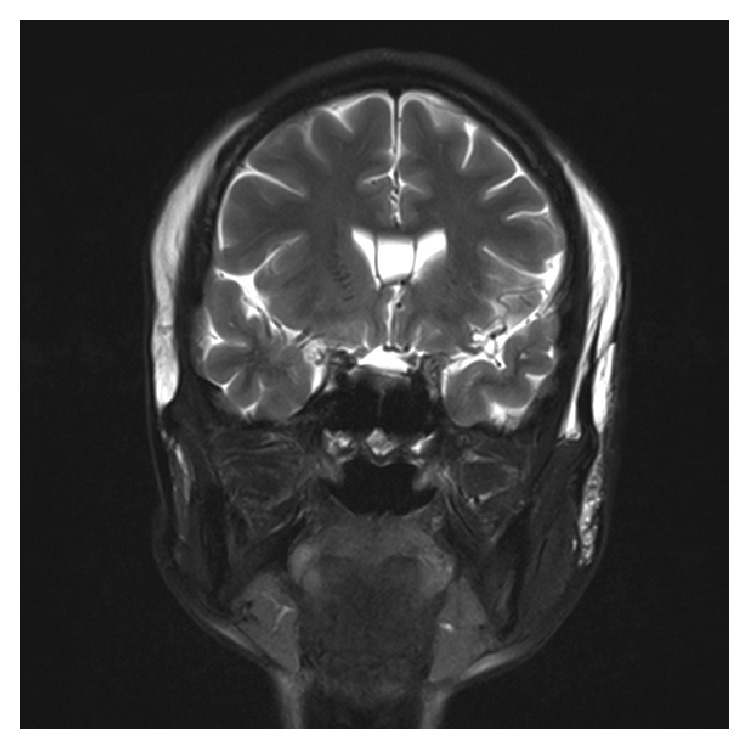
Coronary MR image showing PAAG migration into the cheeks and neck.

**Figure 2:**
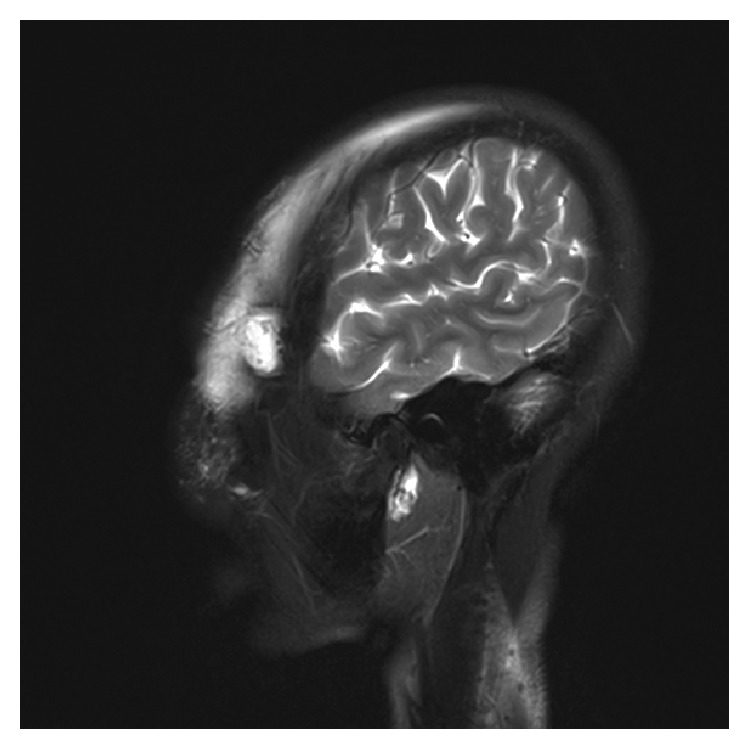
Sagittal image showing PAAG migration into both the upper and lower eyelids.

**Figure 3:**
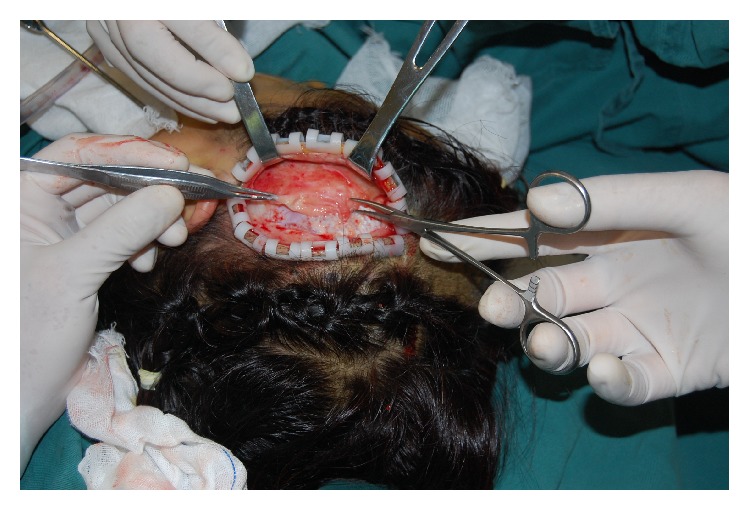
Photograph from the surgery showing the thickened epicranial aponeurosis containing PAAG.

When the patient came to our department 15 years after the first operation, she complained of recurrent swelling of the temporal region, forehead, and eyelids as well as pain at the temporal regions that became even more intense with cold ambient temperature or with large facial expressions. Firm palpation of the temporal region of the scalp over the injected PAAG revealed persistent indention resembling pitting edema, which disappeared after about a minute. Magnetic resonance imaging (MRI) T2 images showed extensive migration of the PAAG as a hyperintense mass. The PAAG had spread from the temple regions to the cheek and neck regions (Figure 1) and from the forehead to both the upper and lower eyelids (Figure 2). The filler had infiltrated the left temporalis more than the right temporalis, which may explain the more intense pain experienced by the patient in the left temporal region compared to the right temporal region. We performed an operation to remove the PAAG under general anesthesia with bilateral temporal incisions in the scalp. When we reached the epicranial aponeurosis, we found it was impossible to separate the PAAG from the patient's tissues because the PAAG had permeated the tissues (Figure 3). Thus, we removed most of the PAAG in the epicranial aponeurosis at the temporal region and forehead together with the affected tissues.

Pathological analysis revealed the presence of an amorphous foreign material deposited in the tissue without any evidence of a foreign body reaction or inflammatory cells. In addition, bacterial cultures were negative. In the routine preoperative examination, the patient's white blood cell count (WBC) was 2.9 × 10^9^/L. However, the patient denied taking any antibiotics or immunosuppression drugs within the previous month. After consultation from the hematologic department, we injected granulocyte colony-stimulating factor subcutaneously the night before the operation, and, in the morning of the operation, the patient's WBC had increased to 14.2 × 10^9^/L. Three months after the operation, the patient returned for follow-up. She reported that the temporal pain and swelling of her forehead and temple were reduced and her WBC was 4.9 × 10^9^/L. However, she refused additional MRI examinations. In the following year, we called the patient to learn whether the leukocytopenia had continued. The patient reported that two additional blood tests carried out since her last follow-up were both normal.

## 3. Conclusions

With the development of biomaterial technology and patients' preferences for less invasive procedures, injection of fillers to change the contour of specific bodily regions has attracted much attention. PAAG was once considered to be a safe filler material and seemed ideal for cosmetic augmentations, especially in the patients suffering from AIDS [2]. However, the use of PAAG as a filler is associated with some troublesome complications, such as PAAG migration, pain, nodule formation, chronic inflammatory reaction, and even skin necrosis and bony erosion [3, 4].

In the present case, the patient received a large injection of PAAG in her epicranial aponeurosis, which brought about a series of complications related to extensive PAAG migration, including recurrent swelling, variable pain induced by temperature changes and facial movement, and leukocytopenia. Compared with previous reported cases of PAAG-related complications, several aspects of the present case should be considered. First, PAAG injection into an inappropriate region may be a principal cause for the complications experienced in this case. To our knowledge, this is the first report of PAAG injection into the epicranial aponeurosis, which is a loose interspace that may potentially promote filler migration into the inferior superficial muscular aponeurotic system. This occurred in this case, with migration not only to the cheek but even to the eyelids and neck. Second, although we cannot be sure that the leukocytopenia in this case was caused by a reaction to the large amount of PAAG injected in this patient, the recurrent swelling may be evidence of chronic inflammation or an autoimmune reaction. Because the histological examination and bacterial culture swab provided evidence of the absence of a foreign body reaction or inflammatory reaction, we can also propose that an allergic or autoimmune reaction rather than a bacterial infection was induced in this case, which is also supported by previous reports [5]. Furthermore, a long-term autoimmune reaction would likely result in immune suppression, leading to leukocytopenia [6]. Third, the temporal pain could have been due to two causes. The first is the degeneration of the temporalis muscle after PAAG injection, which is supported by the previous observation of pectoral muscle degeneration induced by PAAG injected into the breasts [7, 8]. Although we did not collect temporalis tissue for histological examination due to our concern that this might injure the facial nerves, the more severe pain in the left temple, where the muscles showed the most filler infiltration, and the pain induced by changes in facial expression may indicate muscle degeneration. Second, a temperature-induced change in the volume of PAAG could be another reason for the variable pain, because even a very small increase in pressure due to such a change could be felt by a patient if a large amount of filler had been injected or if the filler had penetrated the patient's tissues. Last but not least, the fact that the fillers were not injected properly was the essential reason to induce this result. Firstly, the fillers should be injected in multiple sessions in multiple layers but not a large amount of fillers in a loose interspace such as the epicranial aponeurosis at one time. Secondly, compared with the subcutaneous tissue, it may not be a good idea to inject many fillers adjacent to or into the muscles which may cause muscle degeneration or even leukocytopenia. Thirdly, a relatively larger amount of filler per session such as 8 mL compared with 2 mL may achieve a satisfactory result earlier and reduce hospital costs; anyway, too many fillers into the face in one stage (such as more than 16 mL, according to the literature) may mean a higher incidence of lumps or some other complications [9–11].

In conclusion, application of a large amount of PAAG to a localized region in the body may result in unexpected complications that are difficult to address. Moreover, in addition to localized complications, based on the case presented in this report, we hypothesize that general complications such as a long-term autoimmune reaction leading to leukocytopenia may also be related to PAAG injection. A new material or a new technology may generate excitement, but application prior to sufficient testing can lead to future problems for patients. Furthermore, proper application of any new material or technology is critical.

## Abbreviations

MRI: Magnetic resonance imaging
PAAG: Polyacrylamide hydrogel
WBC: White blood cell count.
